# From Prone to Prepared: Airway Management in a Patient With Penetrating Thoracic Trauma

**DOI:** 10.7759/cureus.76193

**Published:** 2024-12-22

**Authors:** André Santos, Beatriz Leal, Francisco Valente

**Affiliations:** 1 Anesthesiology, Unidade Local de Saúde de São José, Lisbon, PRT; 2 Anesthesiology, Instituto Português de Oncologia de Lisboa Francisco Gentil, Lisbon, PRT

**Keywords:** critical airway, difficult airway management, penetrating thoracic trauma, prone intubation, trauma airway management

## Abstract

Perioperative and critical care management following penetrating thoracic trauma represents a complex challenge. Those who survive the early trauma approach and reach the hospital alive often remain in critical condition, with cardiocirculatory complications and major pulmonary injuries. Additional difficulty arises from the presence of a weapon *in situ*, particularly in a dorsal location, which limits patient positioning, and the safe manipulation of both the weapon and the patient. We present the case of a 47-year-old man, who suffered a stabbing assault, resulting in a deep dorsal thoracic wound with the knife still *in situ*. The patient was initially treated by the pre-hospital team, where the weapon was stabilized with gauze pads and medical tape, and resuscitation was initiated. He was then transported to a regional hospital hemodynamically unstable, requiring further resuscitation with blood products. After stabilization, a computed tomography scan revealed bilateral hemopneumothoraces and the tip of the knife lodged in the lower lobe of the left lung. The hemopneumothoraces were drained and the patient was transported to our trauma center in the prone position, spontaneously breathing with the weapon *in situ*. The patient was proposed to undergo thoracic surgery, specifically an exploratory thoracotomy in the right lateral decubitus position. Airway approach plan A involved anesthetic induction in the prone position while maintaining spontaneous ventilation and placement of an AuraGain™ (Ambu, Denmark) laryngeal mask airway (LMA), followed by fiberoptic guided intubation through the device. Due to glottic edema and inability for glottic progression of the fibrescope, the AuraGain*®* LMA was replaced by an iGel*®* (Intersurgical, UK) LMA, and fiberoptic-guided intubation was successfully achieved. After surgery, the patient remained in the intensive care unit and was successfully extubated five days later. We acknowledge that alternative solutions could have been applied to this case, and we discuss some of them further in this text. This case highlights that, in such complex scenarios, clinical experience and comprehensive knowledge of various airway management devices are critical. Nevertheless, certain principles remain universal in difficult airway management, including the preservation of spontaneous ventilation and meticulous but flexible planning.

## Introduction

Thoracic injuries account for 20-25% of all trauma-related deaths during the first four decades of life [1,2]. Penetrating thoracic trauma can result in a wide range of injuries depending on the entry point, trajectory, and mechanism of injury. The proximity to major intra-thoracic vascular structures and organs can greatly increase the risk of life-threatening complications. The presence of a weapon lodged in the thoracic cavity, especially when the patient is positioned prone, may risk further injury with any manipulation. While massive lung or cardiac damage is often rapidly fatal, less severe lesions allow time for diagnosis and treatment [3]. In those hemodynamically stable, a CT scan is fundamental to evaluate the resultant damage and has a 94% sensitivity in diagnosing tracheobronchial injuries [4,5]. This imagiological evaluation also contributes to planning of airway management, predicting the implications of foreign object removal, and making a team-based decision on the optimal timing for retrieval.

Airway management is a central theme in the practice of anesthesiology, emergency medicine, and the care of critically ill patients in complex circumstances. International guidelines for the management of anatomically difficult airways are well established [6,7], and recent advancements have also provided guidance for the management of physiologically difficult airways [8,9]. Trauma represents a unique scenario where both anatomical and physiological challenges often converge, creating a particularly demanding context. However, there are currently no comprehensive guidelines or recommendations addressing the combination of these factors, and even less guidance exists for rare and highly specific situations, such as the case here presented.

Despite the fact that these guidelines [6-8] cannot be directly or easily applied to complex trauma cases, a key message emerges: in the face of complexity, preserving spontaneous ventilation and fiberoptic-guided intubation remain the cornerstones of safety [4].

Fiberoptic-guided intubation can be performed under varying levels of sedation via a laryngeal mask airway (LMA). In fact, there is some evidence that combining the LMA with fiberoptic guidance leads to higher first-pass success compared with fiberoptic guidance alone [10]. Under that light, the most recent ASA guidelines suggest considering combined techniques in the face of anticipated or unanticipated difficulty [6]. There is limited evidence from comparative studies assessing the ease and success rates of intubation across different types of LMAs. However, it seems intubation with fiberoptic guidance is faster with second-generation LMAs compared with first-generation ones [11]. Additionally, some evidence suggests that the AuraGain™ LMA may outperform the Fastrach™ LMA in terms of glottic visualization, laryngeal mask insertion time, and endotracheal tube (ETT) placement time [12]. It is worth mentioning that this study was performed in anesthetized patients after muscle relaxation. In mannequin studies, there is some indication that iGel® LMA offers greater ease of use compared to the AuraGain™ LMA, though this evidence has not yet been substantiated in human studies [13].

To conclude, out-of-the-box and open-minded anesthetic strategies, combined with the specialized and tailored use of airway equipment, are essential to ensure patient safety, prevent further trauma, and facilitate surgical intervention.

## Case presentation

**Figure 1:**
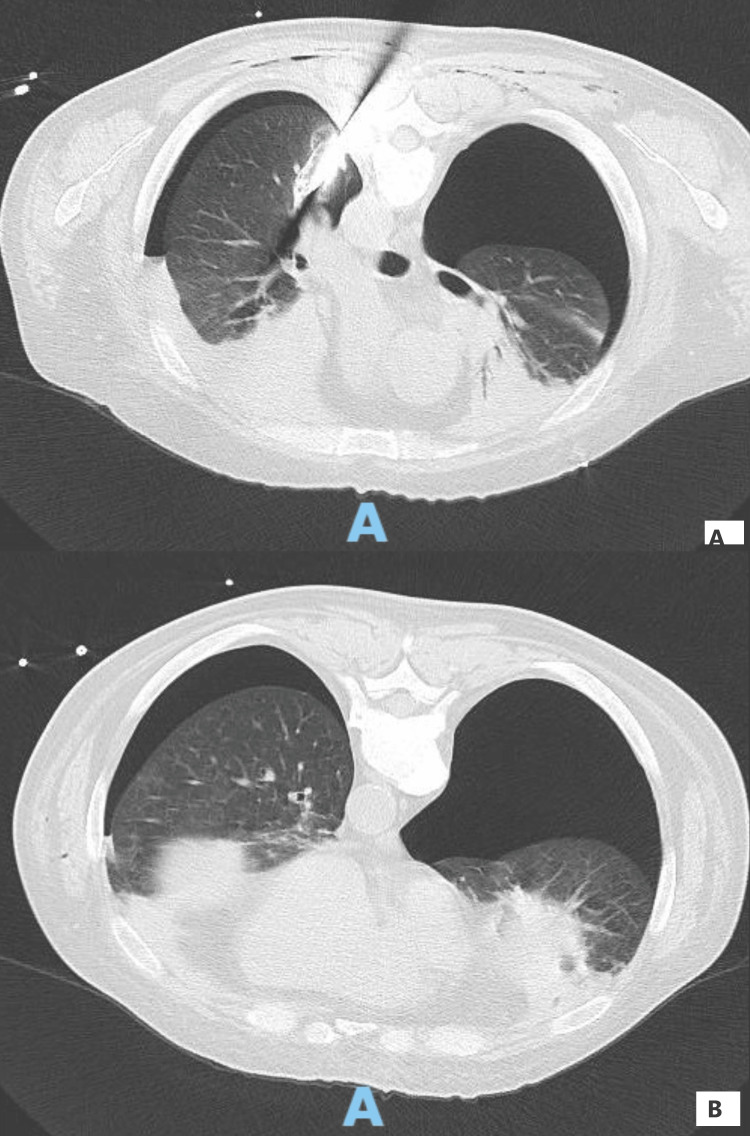
Thoracic CT scan In the first frame, we can see the knife penetrating the lower lobe of the left lung; in the second frame, we can clearly see a large right-sided hemopneumothorax and a moderate left-sided hemopneumothorax. "A" stands for anterior.

We present a case of a 47-year-old man who sustained multiple superficial stab wounds and a dorsal right paravertebral penetrating injury, with the weapon still in situ at hospital arrival (estimated size of the knife of 12-15cm, trajectory anteriorly and to the left). Initial management was provided by the pre-hospital emergency team who stabilized the weapon, started resuscitation with crystalloids, and administered 1 g of tranexamic acid. Upon admission to a peripheral hospital, the patient was in hemorrhagic shock, presenting with a mean arterial pressure of approximately 50 mmHg and a hemoglobin level of 5.2 g/dL (normal range: 11.4-17.5). He required further resuscitation with two units of packed red blood cells, two units of fresh frozen plasma, additional crystalloids (total 4000mL), and transitory vasopressor support, before achieving hemodynamic stability. He was conscious, cooperative (Glasgow coma scale 15), eupneic, and breathing spontaneously on a Venturi mask with oxygen at 15L/min. Arterial blood gas analysis was pH 7.33 (normal range: 7.35-7.45), pCO₂ 47 mmHg (normal range: 32-48), pO₂ 115 mmHg (normal > 80), HCO₃⁻ 23.5 mmol/L (normal range: 22-32), lactate 2 mmol/L (normal < 1.5), and sO₂ 95% (normal range: 94-98). A CT scan subsequently demonstrated a large right-sided hemopneumothorax, moderate left-sided hemopneumothorax, and the knife penetrating the pulmonary parenchyma with the tip at the left lower lobe, proximal to the left ventricle, but without evidence of esophageal or major vascular injury (Figure 1). The hemopneumothoraces were drained by bilateral chest drains, releasing approximately 1000 mL of hematic fluid.

The thoracic surgery team was consulted for further surgical management, and the patient was transferred to our tertiary hospital in the prone position, maintaining spontaneous ventilation, with the knife stabilized in situ to prevent further injury to adjacent structures.

**Figure 2:**
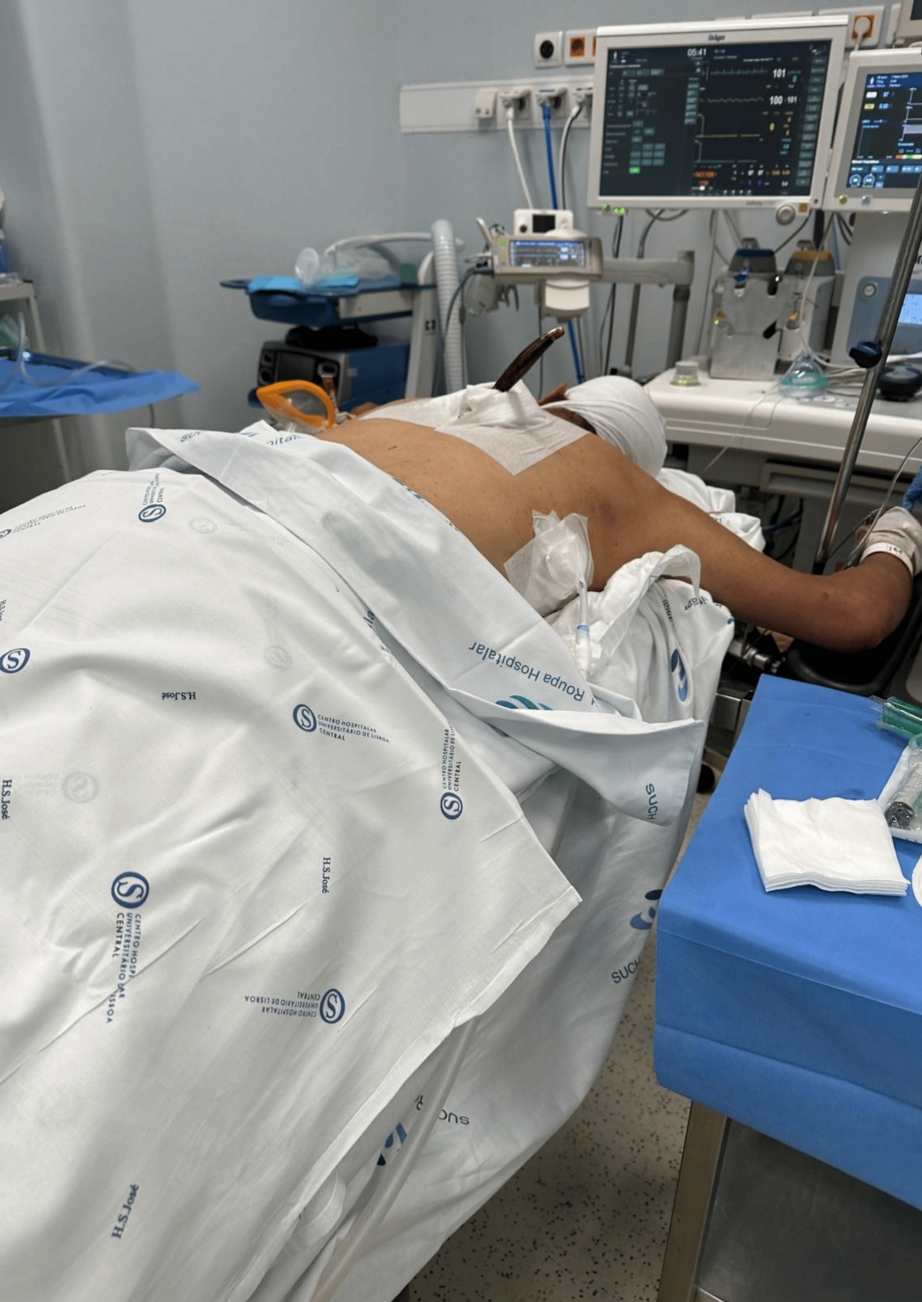
Patient admission to the OR Patient after OR admission, already monitored. The knife was *in situ *and the previously placed chest drains are visible.

At admission (approximately 5 a.m.) he was clinically stable but evidencing discomfort and agitation. Notable facial and periorbital edema was identified, likely secondary to fluid overload and prolonged prone position. Preparations were made for the operating room transfer (Figure 2).

An anesthetic plan was developed with the surgical team to secure the airway while minimizing the risk of mobilization and vascular injury (exploratory thoracotomy to be performed in right lateral decubitus).

Anesthetic management

A stepwise anesthetic strategy was devised with three potential plans, involving all senior professionals from the OR present at that time.

Plan A

Sedation in the prone position while maintaining spontaneous ventilation. Drugs included fentanyl, ketamine, and propofol on demand. An LMA (AuraGain™) was to be placed, followed by fiberoptic-guided intubation through the device. Alternative types of LMA were also available.

Plan B

In the event of failure or complications during Plan A, endotracheal intubation using videolaryngoscopy in prone (ready to use inside the room) while maintaining spontaneous ventilation.

Plan C

If Plans A and B were unsuccessful, immediate reposition of the patient in the right lateral decubitus position, and surgical airway established by the thoracic surgery team (material open and available).

**Figure 3:**
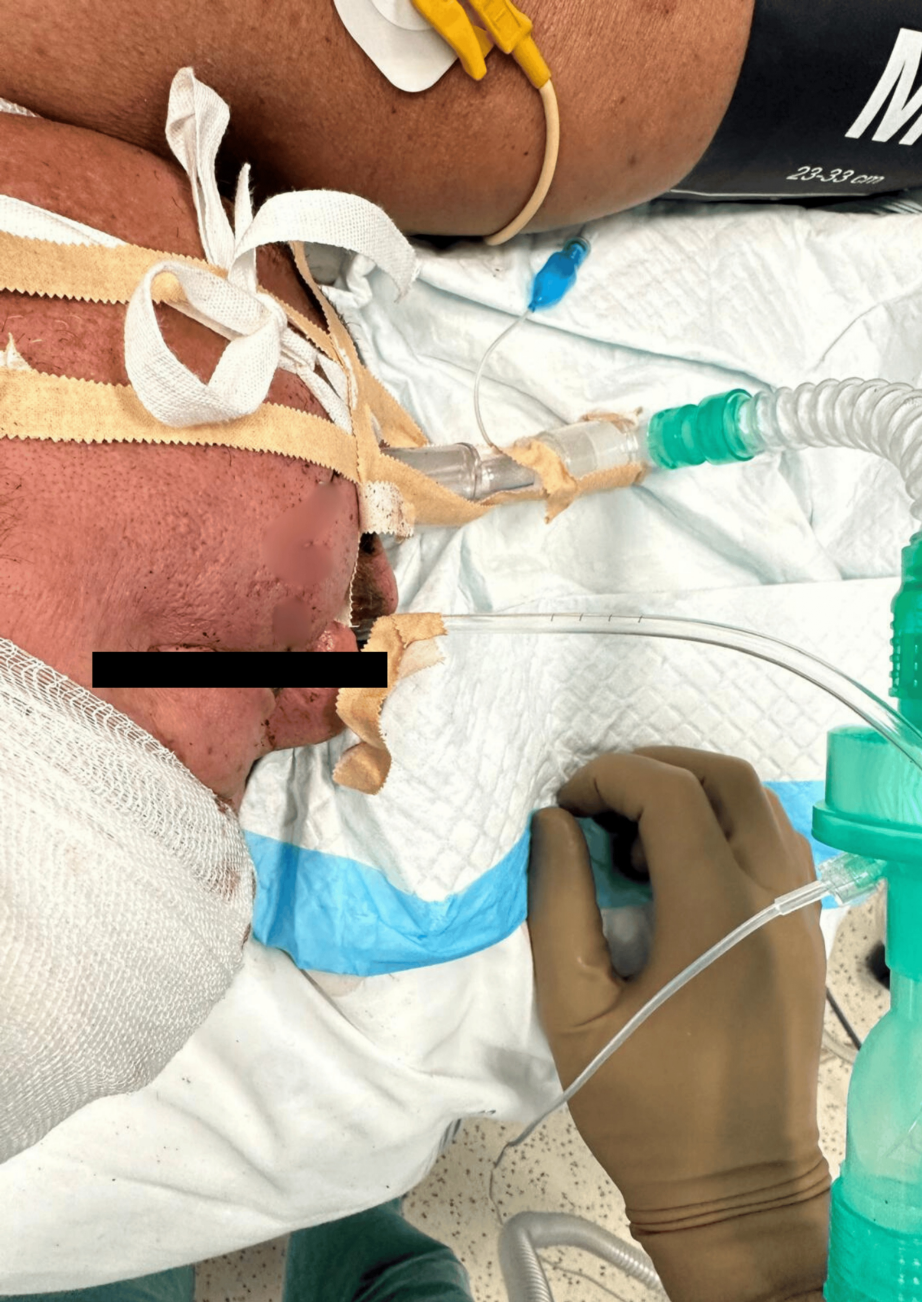
Patient after laryngeal mask and endotracheal tube placement

During the execution of Plan A, the Venturi mask was removed and a well-fitted face mask was adapted, delivering FiO₂ at 100% with an adequate capnography waveform. Subsequently, sedation was performed using approximately 1 mg/kg of ketamine and 1 µg/kg of fentanyl, while maintaining spontaneous ventilation. Following sedation, AuraGain™ LMA was inserted, with good placement and adequate capnography waveform. Additional small boluses of ketamine and propofol were administered to optimize tolerance during airway instrumentation. We must emphasize that spontaneous ventilation was always guaranteed. Difficulty was encountered with the progression of the fiberoptic bronchoscope through the AuraGain™ LMA (despite adequate ventilation with this mask). The AuraGain™ LMA was replaced with an iGel® LMA size 4, and fiberoptic endoscopy was attempted again. The procedure was complicated by glottic edema (probably multifactorial), but successful progression allowed for the placement of a 6.5-mm ETT (Figure 3), without oxygen desaturation.

**Figure 4:**
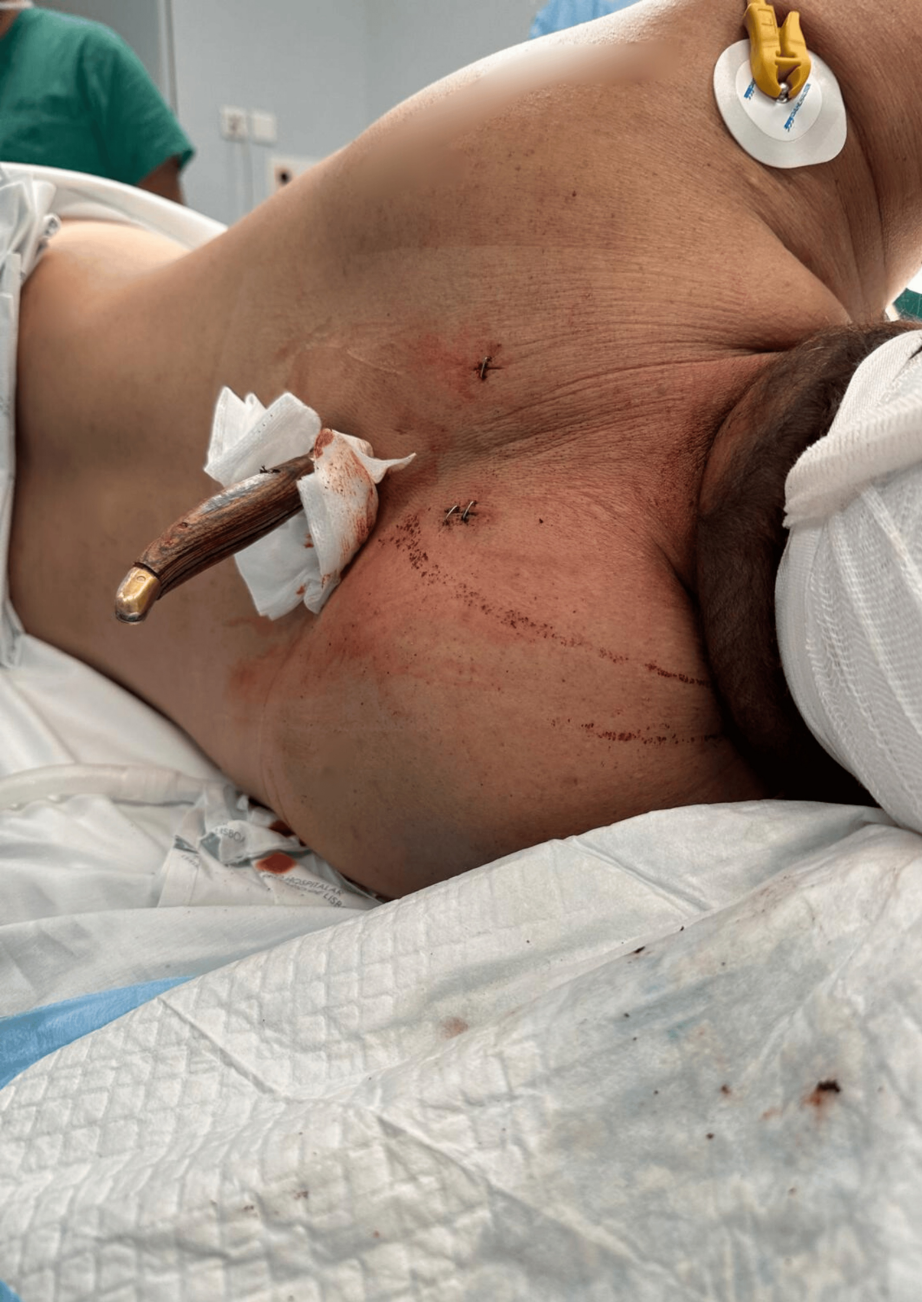
Patient positioned for surgery in the right lateral decubitus

**Figure 5:**
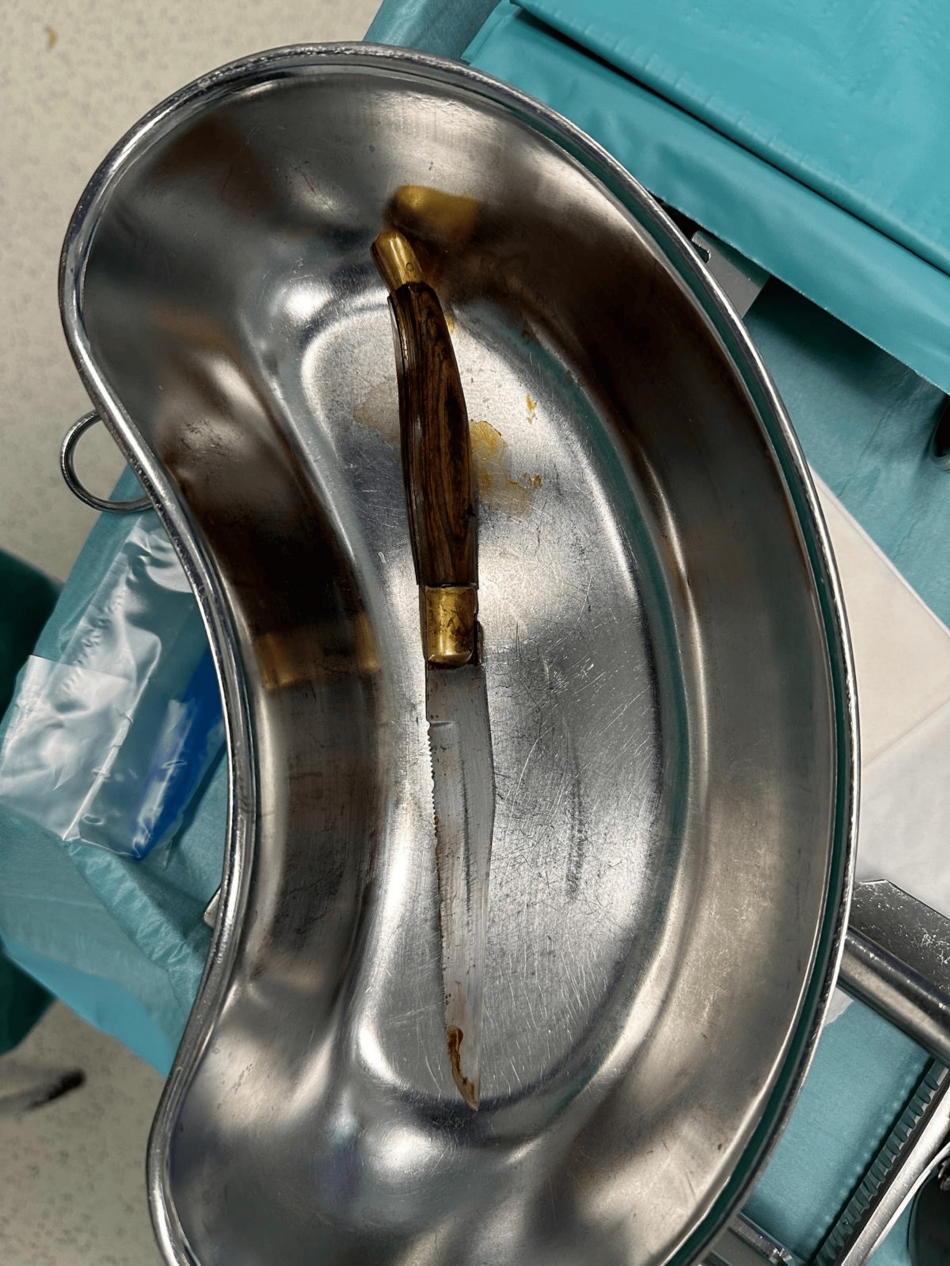
Foreign body (knife)

Following successful intubation, a radial artery line was placed before carefully repositioning the patient to the right lateral decubitus (Figure 4), with hemodynamic and ventilatory stability. Thoracotomy was then performed, and thoracic surgery involved exploration of the wound, extraction of the foreign body (Figure 5), and lung suture. Hemostasis was achieved, new bilateral chest tubes were placed, and the wound was closed. During surgery, in permanent communication with the surgical team, we used small tidal volumes to minimize the risk of additional lung injury caused by the foreign body and to optimize surgical exploration. Upon the extraction of the foreign body, we applied brief periods of apnea to allow for the safe mobilization of the knife.

At the end of the surgery, the 6.5-mm ETT was replaced through an Aintree Intubation Catheter (Cook® Medical, USA) with a 7.5-mm ETT, under videolaryngoscopy in the supine position to facilitate ventilation in the ICU. Extubation was delayed due to evident glottic edema.

The patient was transferred to the ICU. The ENT team did a nasopharyngolaryngoscopy which showed the airway edema and reconfirmed the risk of early extubation. The patient was successfully extubated five days after surgery under high-dose corticotherapy and transferred back to the peripheral hospital eight days after surgery without further complications.

## Discussion

We present the case of a critically injured trauma patient with a knife impaled in the back, penetrating the thoracic cavity near vital structures. This case explores airway management in a prone patient facing ventilatory and hemodynamic challenges.

Medical care organizations tend to concentrate specialized services, such as thoracic surgery, in central hospitals. This often translates to prolonged patient transfers to tertiary care centers equipped with these capabilities, adding logistical and clinical challenges. Although trauma centers are recognized as reference institutions, with experienced professionals and advanced tools, the timing of patient admission can be a limiting factor. Admission during certain hours may impact the availability of resources, the freshness of the team, and access to critical specialties.

Airway management in trauma is particularly challenging, and, as mentioned previously, there are no specific guidelines or recommendations for such cases. To the best of our knowledge, there are very few documented cases addressing airway management in a prone patient with a dorsal weapon retained* in situ*. Interestingly, none report lung trauma, nor our airway approach using LMA and fiberoptic guidance to intubate [14-17].

Despite the rarity of the case, we committed to golden rules commonly used in airway management. These included maintaining spontaneous ventilation as a cornerstone of safety, establishing multiple airway plans, ensuring the availability of a wide range of equipment to address unforeseen complications, and involving a multidisciplinary team to align strategies and foster coordinated care.

In our approach, topicalization of the airway with local anesthesia could have been performed to improve patient comfort and suppress airway reflexes during the procedure, especially in the context of sedation. The outlined airway plans culminated in a surgical airway as a last resort. In a critical situation, it could also have been considered the removal of the weapon to attempt intubation in the supine position (Plan D). Given the unpredictability of the case, more than one plan should have been defined to address the worst-case scenario.

The decision to perform fiberoptic intubation was dictated by the impossibility of employing other airway techniques due to the patient’s positioning and the weapon *in situ*. Given the patient’s agitation, and likely inability to remain still during the procedure, LMA was selected to facilitate the technique under adequate sedation. Among the available LMAs, the Ambu®AuraGain™ was initially chosen based on team expertise, and evidence supporting its effectiveness [12].

## Conclusions

Based on our emergency care experience and the existing literature, cases involving prone patients with a weapon deep in the upper back and severe thoracic trauma are rare. The main goal of this article is to present our airway management approach and discuss our options. While some aspects of our technique were guided by available evidence, many decisions had to be made without such support. Nonetheless, our approach relied greatly on non-technical skills, such as situational awareness and effective teamwork, which are increasingly recognized as fundamental components in the management of complex scenarios. Certainly, alternative solutions could have been proposed for this case. Different teams in different realities could have taken different paths. Nevertheless, the key points to ensure technical success and patient safety would have to be the same: careful team planning, anticipation of potential challenges, establishing priorities, and defining innovative and thoughtful strategies using a deep knowledge of airway equipment.
